# Social Adversity, Sleep Characteristics, and Elevated Blood Pressure Among Young Adult Black Females

**DOI:** 10.1089/heq.2020.0033

**Published:** 2020-10-07

**Authors:** Jewel Scott, Susan Silva, Leigh Ann Simmons

**Affiliations:** ^1^ Duke University School of Nursing, Durham, North Carolina, USA.; ^2^ Department of Human Ecology, University of California, Davis, Davis, California, USA.

**Keywords:** sleep, hypertension, discrimination, health disparities, young adulthood, stress

## Abstract

**Purpose:** We examined whether sleep characteristics and adverse social exposures were associated with elevated blood pressure (BP) in young adult black women.

**Methods:** This is a cross-sectional analysis of existing data from 581 black females who participated in the National Longitudinal Study of Adolescent to Adult Health (Add Health). Adverse social exposures included child abuse, discrimination, perceived stress, social isolation, and subjective social status. Self-reported sleep characteristics were measures of duration, latency, continuity, and snoring. Logistic regression was used to evaluate the influence of social exposures and sleep characteristics on BP.

**Results:** Among the women (mean age=29.1 years), 32.4% had elevated BP (≥130 systolic or ≥80 diastolic). In adjusted analysis, poor sleep continuity (adjusted odds ratio [aOR]=1.70, 95% confidence interval [CI]=1.07–2.70) and discrimination (aOR=1.61, 95% CI=1.00–2.58) were associated with higher odds of elevated BP, while more social isolation (aOR=0.69, 95% CI=0.48–0.99) was associated with lower odds of elevated BP.

**Conclusion:** Poor sleep continuity and experiencing discrimination may represent key risk factors for hypertension in young black females. Unexpectedly, being more isolated was associated with lower BP. Future research should examine how to adapt current paradigms and measures of social connectedness, isolation, and stress to better elucidate the impact of these factors on the long-term health of young black females.

## Introduction

Hypertension is among the top three leading contributors to death and disability worldwide,^1^ and black females are among the most affected. In the United States, the overall prevalence of hypertension is 29%, but among black females, the prevalence is 40% (Ref.^2^). Elevated blood pressure (BP) develops at younger ages in black females compared to women of other ethnicities.^1,3^ More attention to risk factors that contribute to elevated BP in black women younger than 40 years is needed.

Known risk factors for elevated BP, such as obesity and family history, do not fully explain the disparity in elevated BP prevalence, especially among young black females.^4,5^ Mounting evidence suggests that adverse social exposures, including child abuse,^5,6^ social isolation,^7,8^ discrimination,^9–11^ and socioeconomic disadvantage,^11–13^ have a negative impact on physical health. For example, in a nationally representative study of over 16,000 individuals, social isolation was identified as a risk factor for mortality with effects on par with tobacco use and BP.^8^ Owing to the social stratification of society, discrimination is a social adversity that many black women experience.^10,11,14^ In the Jackson Heart Study, a prospective, cohort study of black Americans, women were more likely to report discrimination as being very stressful, and the burden of discrimination was associated with high BP.^14^ Current and early life experiences of adversity are potential contributors to adult cardiovascular health (CVH). Exposure to multiple forms of childhood adversity, such as child abuse and parental mental illness, is associated with up to a fourfold increase in risk for cardiovascular disease (CVD).^5,6^ Despite these documented relationships, few studies have focused on these stressors exclusively in young black women.

Sleep as a health behavior is important, because a growing body of literature has identified a strong association between sleep and CVD.^15^ Insomnia (i.e., difficulty falling asleep or staying asleep) and short sleep duration are examples of sleep characteristics that elevate risk for hypertension,^15^ but have not been widely studied among young black women. Researchers using data from the National Longitudinal Youth Survey found a divergence in sleep duration between white and black young adults occurs around age 24 years, after which black young adults sleep less.^16^ Examining sleep behaviors during young adulthood could be critical for early identification of black women at risk for hypertension.

Comparative studies were instrumental in identifying inequities in CVH that exist between populations in the United States. However, now that CVH disparities are well established, within-group studies may permit a more contextualized study of social contributors to the development of CVH in black women as supported by critical race theory.^17^ Furthermore, the weathering hypothesis underscores the need to study whether adversities and health behaviors are associated with BP in young adult black women.^18,19^ Thus, the research question is, “Are multiple social adversities and sleep characteristics indicators of increased risk for elevated BP among young Black females?”

## Methods

### Design and sample

**FIG. 1.**
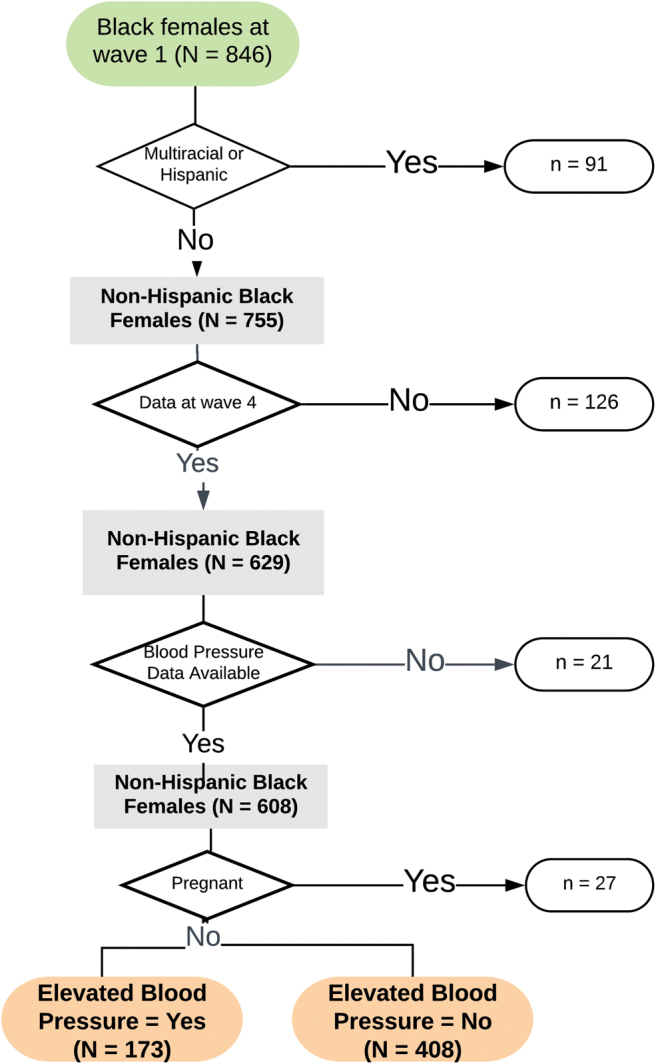
Data available for analysis.

This study was a secondary analysis of data collected as part of The Longitudinal Adolescent to Adult Health Study (Add Health)^20^ and deemed exempt by the Institutional Review Board. A total of 20,745 students were enrolled at wave 1 in 1994, and followed prospectively with periodic, in-person data collection. The study has maintained an overall retention rate of 80.3%. Data for this project were obtained from the Add Health fully de-identified, public use data, which is a random sampling of 50% of the total study population. The analysis sample comprised non-Hispanic, black females who participated in the 2006–2008 (wave 4) data collection when BP was assessed for the first time. Females who identified black as one of multiple racial identities were excluded because social experiences and health outcomes may be meaningfully different for multiracial females.^21^ Women who were pregnant were also excluded because physiological changes in pregnancy can result in altered BP.^22^ The analysis included 581 women who met eligibility criteria (Fig. 1).

### Measures

#### Outcome

The outcome was elevated BP, as defined by the 2017 American Heart Association (AHA) guidelines. Participants rested in a seated position for 5 min before three BP measurements were collected using the Microlife BP3MC1-PC-IB monitor (MicroLife USA, Dunedin, FL).^23^ The first reading was discarded and the average of the second and third measurements was recorded. Consistent with the most recent guidelines, systolic BP ≥130 or a diastolic BP ≥90 was coded as elevated BP. Since BP is only measured once, we cannot determine hypertensive status, but as BP readings increase, cardiovascular (CV) risk also increases.^1,24^ Thus, a single BP reading can provide meaningful data about potential risk for early-onset CVD in a young adult population. Covariates included self-reported prior diagnosis of hypertension, diabetes, hyperlipidemia, and heart disease.

#### Demographic and clinical characteristics

Age, education, and annual income were self-reported. Height and weight were measured and overweight and obesity were determined by applying the AHA standardized cutoffs.^1^ Depression symptoms were measured using the 10-item Center for Epidemiologic Studies Scale, with scores of 10 or above representing clinically significant depressive symptoms.^25,26^ The weighted Cronbach's α in this sample was α=0.82. Participants rated their overall health, and from this, a dichotomous measure (poorer health, defined as fair/poor health vs not poorer health, defined as good/very good/excellent) was created, consistent with other research of self-rated health.^27^

#### Sleep characteristics

The following four sleep characteristics were assessed at wave 4: (1) snoring, (2) sleep continuity, (3) sleep onset, and (4) sleep duration. The first three were assessed as dichotomous questions, whereas sleep duration was assessed based on self-reported bed and wake times on a typical work or school day. See Table 1 for definitions and coding.

**Table 1.** Participant Characteristics (*N*=581)

Predictor	Description	n^a^	Weighted	
			Mean or percent	SE
Demographics				
Age	Age in years	581	29.09	0.22
Low education	Yes (≤high school)	116	25.91%	3.40
	No (post-secondary)	465	74.09%	3.40
Low income	Yes (<20 K)	140	31.51%	3.10
	No	397	68.49%	3.10
Clinical characteristics				
Overweight/obese	Yes (BMI ≥25)	436	77.68%	1.87
	No	140	22.32%	1.87
Depression symptoms	Yes (CESD ≥10)	147	26.51%	2.19
	No	434	73.49%	2.19
Poorer health	Yes (self-rated fair, poor)	82	15.06%	1.85
	No	499	84.94%	1.85
Health behaviors				
Tobacco use	Yes	141	26.43%	3.37
	No	429	73.57%	3.37
Poor diet	Yes (fast food >2 days/week)	237	42.06%	2.38
	No	342	57.94%	2.38
Physical inactivity	Yes (<4 days a week)	491	86.72%	1.61
	No	90	13.28%	1.61
Sleep characteristics				
Delayed sleep onset	Yes (difficulty falling asleep)	189	34.31%	2.19
	No	388	65.69%	2.19
Poor sleep continuity	Yes (frequent awakening)	229	40.03%	2.43
	No	347	59.97%	2.43
Snoring	Yes (snore/stop breathing)	283	51.43%	2.82
	No	291	48.57%	2.82
Short sleep duration	Yes (<6 h)	62	11.00%	1.57
	No	518	89.00%	1.57
Long sleep duration	Yes (>9 h)	54	9.56%	1.32
	No	526	90.44%	1.32
Adverse social exposures				
Social isolation	Yes	339	57.49%	3.33
	No	242	42.51%	3.33
Discrimination	Yes	171	31.13%	2.40
	No	410	68.87%	2.40
High perceived stress	Yes (PSS-4 above 5)	338	59.51%	3.45
	No	243	40.49%	3.45
Low subjective social status	Yes	402	71.16%	2.47
	No	176	28.84%	2.47
Child abuse	Yes	184	31.43%	1.98
	No	382	68.57%	1.98
Outcome: elevated BP	Yes (BP ≥130/90)	173	32.38%	2.60
	No	408	67.62%	2.60

^a^ Unweighted.

BMI, body mass index; BP, blood pressure; CESD, Center for Epidemiologic studies; PSS, perceived stress scale; SE, standard error.

#### Adverse social exposures

Adverse exposures included the following: (1) discrimination, (2) subjective social status, (3) perceived stress, (4) social isolation, and (5) child abuse. Discrimination was measured as a single question from the Everyday Discrimination Scale,^11^ which asked “How often are you treated with less courtesy or respect than others?” Responses were dichotomized such that often or frequent were coded as yes. Subjective social status was assessed using the MacArthur scale, which is based on an ordinal, 10-step scale where 10 represents people with the most money, education, and best respected jobs.^12,13^ Using a picture of a 10-step ladder, participants were asked to indicate their social position compared to other people in the United States. Perceived stress was measured using the 4-item Perceived Stress Scale,^28^ and the weighted Cronbach's α in this sample was 0.64. Both subjective social status and perceived stress were dichotomized based on their respective medians to facilitate a parsimonious analysis of these continuous variables. Social isolation was assessed as a dichotomous question, which asked “How often do you feel isolated from others?” Child abuse was assessed retrospectively at wave 4, using questions such as “before age 18 years, how often did a parent or caregiver hit you with a fist, or kick you?” Any physical, emotional, or sexual abuse constituted an adverse social exposure (yes/no).

#### Health behaviors

Participants were asked if they had used tobacco in the last 30 days, and any use was coded as yes. Diet was assessed by the question, “How many times did you eat fast food in the last 7 days?” Fast food intake of more than twice per week was coded as yes for unhealthy diet, based on other studies of dietary habits and CVH.^29^ Physical activity was assessed with a series of questions about frequency of participation in a variety of activities (e.g., dance and aerobics) in the last 7 days. The activity frequencies were summed to represent activity level of participants. Fewer than four occurrences of activity in the last 7 days was coded as inactive.

### Analytic strategy

Data were analyzed using SAS version 9.4 (SAS Institute, Cary, NC). Sample weighting to account for the complex survey design was applied in all analyses to ensure representativeness of the survey data. Descriptive statistics were used to detail sample characteristics and analysis variables. Nondirectional statistical tests were conducted with significance set at 0.10 per test for bivariate analyses to select eligible covariates for the multivariable analysis, where significance was set at 0.05. The significance level was not adjusted for multiple tests as the intent of this initial exploratory study was to inform the development of future hypothesis-driven research.^30^

Bivariate logistic regression was used to examine the relationship between each sleep characteristic and adverse social exposure with elevated BP. In addition, bivariate regression was applied to identify demographic and clinical characteristics, and health behaviors significantly associated with elevated BP. The latter characteristics were included as covariates in the subsequent multivariable regression model.

The initial multivariable logistic regression included sleep characteristics, and adverse social exposures as predictors of elevated BP (model 1). Model 2 adjusted for demographics, clinical characteristics, and health behaviors and model 3 adjusted for all of the above and past medical history. Odds ratios (ORs) from the bivariate models and adjusted odds ratios (aORs) from the multivariable models along with their 95% confidence intervals (CIs) were used to address effect size and clinical relevance of results. The sample size of 581 provided at least 80% power for the initial and most complex multivariable model with potentially 20 predictors (demographic, clinical, health behavior, sleep, and adverse social exposures).

## Results

### Participant characteristics

Table 1 presents the characteristics of the 581 young adult black females, of whom 408 had normal BP (elevated BP=0, no) and 173 women had elevated BP (elevated BP=1, yes). The mean age was 29.1 years (range: 25–34 years) with 32% with a low annual income. Clinically, 78% had overweight or obesity, 27% reported depressive symptoms, 15% rated their health as poor or fair, 26% used tobacco products, and 87% were physically inactive. Of the sleep characteristics evaluated, snoring (51%) followed by poor sleep continuity (40%) and delayed sleep onset (34%) were the most common sleep disturbances. The most commonly reported adverse social exposures were low subjective social status (71%), high perceived stress (60%), and social isolation (57%).

### Bivariate analysis

In bivariate analysis, two of the five sleep characteristics were significant predictors of elevated BP (Table 2). The odds of elevated BP were significantly higher among those who reported the following: (1) snoring or being told that they stopped breathing when asleep (OR=1.72, 95% CI=1.21–2.45) and (2) poor sleep continuity (OR=1.53, 95% CI=1.03–2.27). For adverse social exposures, the odds of elevated BP were significantly higher among those who reported discrimination (OR=1.90, 95% CI=1.30–2.78), but was significantly *lower* among those who reported child abuse (OR=0.56, 95% CI=0.38–0.84). Next (*not shown*), we examined type of child abuse (i.e., physical, sexual, and emotional) separately as predictors. The results were mostly consistent with the above results, so subsequent analyses were conducted using the combined abuse variable.

**Table 2.** Bivariate Relationships Between Predictors and Blood Pressure (*N*=581)

Predictor		Elevated BP	Weighted			p
		Present, n (%)	SE	OR	95% CI	
Demographics						
Age (descending)		—	—	1.14	1.01–1.28	**0.033**
Low education	Yes	44 (39.32)	5.26	1.52	0.94–2.44	**0.086**
	No (Ref.)	129 (29.94)	2.71			
Low income	Yes	44 (33.17)	5.05	1.03	0.64–1.66	0.916
	No (Ref.)	115 (32.61)	2.85			
Clinical characteristics						
Overweight/obese	Yes	157 (36.84)	2.78	2.96	1.39–6.31	**0.005**
	No (Ref.)	14 (16.44)	5.00			
Depression symptoms	Yes	41 (30.19)	4.79	0.87	0.52–1.47	0.602
	No (Ref.)	132 (3.16)	3.04			
Poorer health	Yes	37 (45.04)	6.22	1.90	1.10–3.27	**0.021**
	No (Ref.)	136 (30.13)	2.76			
Health behaviors						
Tobacco use	Yes	39 (32.08)	4.36	0.98	0.59–1.65	0.951
	No (Ref.)	130 (32.43)	3.40			
Poor diet	Yes	74 (32.27)	3.90	0.99	0.60–1.64	0.965
	No (Ref.)	98 (32.51)	3.75			
Physical inactivity	Yes	141 (31.93)	2.98	0.86	0.47–1.58	0.624
	No (Ref.)	32 (35.30)	5.99			
Sleep characteristics						
Delayed sleep onset	Yes	78 (40.2)	4.70	1.36	0.90–2.03	0.142
	No (Ref.)	126 (33.2)	2.41			
Poor sleep continuity	Yes	93 (41.5)	4.23	1.53	1.03–2.27	**0.035**
	No (Ref.)	111 (31.6)	2.67			
Snoring	Yes	118 (41.7)	3.27	1.72	1.21–2.45	**0.003**
	No (Ref.)	85 (29.3)	3.12			
Short sleep	Yes	29 (47.8)	7.56	1.74	0.92–3.31	0.090
	No (Ref.)	177 (34.5)	2.56			
Long sleep	Yes	24 (37.9)	8.30	1.10	0.52–2.34	0.809
	No (Ref.)	182 (35.7)	2.70			
Social exposures						
Social isolation	Yes	111 (33.1)	3.05	0.76	0.55–1.06	0.103
	No (Ref.)	95 (39.4)	3.21			
Discrimination	Yes	75 (46.2)	4.09	1.90	1.30–2.78	**0.001**
	No (Ref.)	131 (31.1)	2.86			
High perceived stress	Yes	120 (34.2)	3.01	0.84	0.55–1.28	0.417
	No (Ref.)	86 (38.2)	4.05			
Low subjective social status	Yes	151 (36.9)	3.09	1.17	0.70–1.95	0.542
	No (Ref.)	54 (33.3)	4.76			
Child abuse	Yes	58 (27.4)	3.71	0.56	0.38–0.84	**0.006**
	No	143 (40.1)	3.00			

Bold indicates *p* < 0.05.

All models are adjusted for the complex sampling design.

CI, confidence interval; HTN, hypertension; OR, odds ratio.

Of the demographics and clinical characteristics, the odds of elevated BP were significantly higher among participants who (1) were older (OR=1.14, 95% CI=1.01–1.28); (2) had lower educational levels (OR=1.52, 95% CI=0.94–2.44); (3) had overweight or obesity (OR=2.96, 95% CI=1.39–6.31); and (4) reported poorer self-rated health (OR=1.90, 95% CI=1.10–3.27). None of the health behaviors significantly predicted elevated BP. Thus, age, overweight/obese, low education, and self-rated health were included as covariates in the multivariable model.

### Multivariable analysis

In the first model (Table 3), poor sleep continuity (aOR=1.64, 95% CI=1.04–2.60), snoring (aOR=1.70, 95% CI=1.13–2.56), and experiencing discrimination (aOR=2.15, 95% CI=1.38–3.33) were significantly associated with higher odds of elevated BP. Social isolation (aOR=0.56, 95% CI=0.36–0.88) and child abuse (aOR=0.53, 95% CI=0.32–0.86) were associated with lower odds of elevated BP. The associations with elevated BP remained significant in model 2, but some were no longer significant in the final model.

**Table 3.** Relationship Between Sleep, Social Adversity, and Blood Pressure (*N*=581)

Domain	Model 1		Model 2		Model 3	
	aOR	95% CI	aOR	95% CI	aOR	95% CI
Sleep characteristics						
Delayed sleep onset	1.16	0.74–1.83	1.17	0.70–1.94	1.09	0.62–1.92
Poor sleep continuity	**1.64**	1.04–2.60	**1.86**	1.16–2.98	**1.70**	1.07–2.70
Snoring	**1.70**	1.13–2.56	**1.51**	1.01–2.24	1.43	1.01–2.04
Short sleep	1.06	0.48–2.33	0.79	0.38–1.64	1.28	0.62–2.66
Long sleep	1.45	0.71–2.95	1.38	0.66–2.89	1.39	0.55–3.50
Social adversity						
Social isolation	**0.56**	0.36–0.88	**0.60**	0.38–0.95	**0.69**	0.48–0.99
Discrimination	**2.15**	1.38–3.33	**1.79**	1.09–2.95	**1.61**	1.00–2.58
High perceived stress	0.65	0.38–1.11	0.68	0.39–1.17	0.66	0.38–1.16
Low subjective social status	1.18	0.66–2.09	1.18	0.66–2.09	1.15	0.68–1.95
Child abuse	**0.53**	0.32–0.86	**0.55**	0.33–0.93	0.53	0.33–0.87
Demographics						
Age (descending)			1.13	0.97–1.30	1.04	0.90–1.20
Low education			1.35	0.75–2.42	1.50	0.79–2.88
Clinical characteristics						
Overweight/obese			**3.25**	1.54–6.88	**3.20**	1.52–6.71
Poorer health			1.31	0.71–2.41	0.94	0.44–2.02
Medical history						
Hypertension					**6.94**	3.35–14.40
Diabetes					0.74	0.18–3.00
Hyperlipidemia					0.47	0.13–1.69
Heart disease					1.59	0.29–8.84

Bold aOR=*p*<0.05.

All models are adjusted for the complex sampling design.

aOR, adjusted odds ratio; Model 1, adjusted for other sleep characteristics and social adversity variables; Model 2, adjusted demographics and clinical characteristics; Model 3, adjusted for past medical history.

In the fully adjusted model, the probability of elevated BP was significantly higher in black women who reported the following: overweight/obesity (aOR=3.20, 95% CI=1.52–6.71), prior diagnosis of hypertension (aOR=6.94, 95% CI=3.3–14.40), poor sleep continuity (aOR=1.70, 95% CI=1.07–2.70), and discrimination (aOR=1.61, 95% CI=1.00–2.58). Interestingly, the adjusted odds of elevated BP remained significantly lower among those who reported more social isolation (aOR=0.69, 95% CI=0.48–0.99).

## Discussion

We examined the association of adverse social experiences and sleep characteristics with hypertension among young adult black females, an age and sex demographic at high risk for hypertension. Our novel findings identified discrimination and poor sleep continuity as significant risk factors for elevated BP among this subgroup of women, highlighting the importance of assessing for a broad range of risk factors beyond the traditional diet and physical activity behaviors. This study is among the few to use population representative data and objective BP data to examine sleep and the social environment as risk factors for young black women to develop hypertension.

Poor sleep continuity, assessed as frequent awakening, was the only sleep characteristic that was significantly associated with BP after adjusting for other factors that influence the development of hypertension. There are many possible causes of poor sleep continuity. We examined two possibilities, snoring, which may be related to sleep apnea, and depression, and neither was significantly associated with hypertension in this sample. However, we did not measure anxiety, which is also associated with both sleep and hypertension.^31,32^ Future research should attempt to replicate our findings, as well as explore other individual and family factors that may contribute to poor sleep continuity. Possibilities include frequent awakening related to a bed partner's snoring,^33^ caregiving of young children, loneliness,^34^ stress,^32^ and traumatic experiences (e.g., nightmares).^35^ Racism and discrimination are examples of chronic stressful experiences, which could impair sleep through rumination^36,37^ and perceived stress,^38,39^ but future work should explore these mechanisms further.

Women who reported experiencing discrimination had almost double the odds of hypertension compared to young women who did not report discrimination. This relationship was only partially attenuated when we controlled for other factors influencing hypertension. Our findings are somewhat consistent with other studies examining the association of discrimination and BP, although few studies have examined this relationship in young adults. A study of 30,000 black women with a median age of 37 years found a positive association between discrimination and hypertension only for women born outside of the United States and those who grew up in predominantly white communities.^40^ Krieger and Sidney^10^ examined the relationship of discrimination and BP in a similar sample of young adults. They found that BP was higher in working class black women who reported no discriminatory experiences, or a passive coping style, such as keeping such experiences to themselves.^10^ Future research should explore the experiences of young adults related to unfair treatment, ways of coping, and potential CV consequences of both discrimination and behavioral responses to discrimination. Clinicians and researchers should also be aware of the impact of social adversities on health to advocate for communities at the macro level and to develop interventions that support positive health behaviors in the face of adversity.

A surprising finding was the inverse association of social isolation with elevated BP. Stated plainly, *more* social isolation was associated with *lower* BP. At first examination, this relationship seems counterintuitive, given studies that suggest social support buffers against stress. However, the wear and tear from the expectations that come with family and social relationships may take a toll on CVH. In fact, the negative health consequences of caregiving are well documented in the literature.^41,42^ Caregiving occurs in the context of raising a family, as is common for the developmental stage of young adulthood, plus some black women have been caregiving since childhood due to phenomena like grandparents raising children, and heart disease and strokes affecting black families at earlier ages.^42^ Consequently, for some black women, the internalization of the “Strong Black Woman” ideology includes a relentless obligation to care for others, sometimes to the detriment of their own health.^43,44^ Understanding how social networks and family life affect young black women is a critical future area for study. Researchers must begin to untangle the nuanced ways that social and family connections, and the expectations that come with them, may serve as protective factors or may impose risk.

### Limitations

This study is among the few to focus on BP in young adult black women, but the cross-sectional design is a significant limitation. A second limitation is the measurement of social adversity factors (e.g., discrimination) using single-item questions; however, all items are from validated instruments. Our findings are useful for identifying areas of focus in future research tailored to address health inequities experienced by young black women.

As this study is a secondary analysis of publicly available data from a large, multistate study, we are limited to the data that are available. Consequently, we were not able to adjust for medication use, but we did adjust for the most common indications for the use of antihypertensive medications. The existing data also do not include prior diagnosis of obstructive sleep apnea, which is a risk factor for hypertension and associated with several of the sleep characteristics examined in this study.^1,15^ Nevertheless, after covarying for CV comorbidities associated with obstructive sleep apnea, poor sleep continuity remained a significant predictor of elevated BP. The findings from this study are a significant contribution to the literature, given the objective measurement of the outcome variable, BP, the population representativeness of our sample, and the inclusion of behaviors and determinants that have not been as well studied in this group of women.

Our assessment of child abuse is a retrospective account, as are most published studies of the CVH consequences of child abuse. However, previous investigations have determined that retrospective reports of child maltreatment are reliable and may be conservative estimates.^45,46^ Finally, all self-reported data are subject to bias, including our measurement of sleep behaviors. Future research should continue to explore BP and sleep behaviors in young black women utilizing objective sleep data, such as polysomnography.

## Conclusion

Adverse social exposures and poor sleep continuity are understudied as risk factors for hypertension in young black females. Based on our findings, risk assessments for this group of women should include questions about social network burden and experiences of discrimination. Collaborative models with community-based behavioral health care providers could facilitate young women receiving the mental health support needed to manage the stress related to social adversities. However, discrimination and other social adversity stem from inequities in the community and society at large; thus, multilevel approaches at the local, community, and national levels are needed.

## Abbreviations Used

AHA: American Heart Association
aOR: adjusted odds ratio
BMI: body mass index
BP: blood pressure
CESD: Center for Epidemiologic Studies
CI: confidence interval
CVD: cardiovascular disease
CVH: cardiovascular health
HTN: hypertension
OR: odds ratio
PSS: perceived stress scale
SE: standard error
